# A repeated cross-sectional analysis assessing mental health conditions of adults as per student status during key periods of the COVID-19 epidemic in France

**DOI:** 10.1038/s41598-021-00471-8

**Published:** 2021-11-09

**Authors:** Melissa Macalli, Nathalie Texier, Stéphane Schück, Sylvana M. Côté, Christophe Tzourio

**Affiliations:** 1 Inserm, Bordeaux Population Health Research Center, UMR 1219, University of Bordeaux, 146 Rue Léo Saignat, 33076 Bordeaux Cedex, France; 2 Kappa Santé, 4 Rue de Cléry, 75002 Paris, France; 3 School of Public Health, University of Montreal, Montreal, QC H3T 1J4 Canada

**Keywords:** Psychology, Risk factors

## Abstract

Previous studies have shown the negative impact of the COVID-19 epidemic on students’ mental health. It is, however, uncertain whether students are really at higher risk of mental health disturbances than non-students and if they are differentially impacted by lockdown periods over time. The objective of our study was to compare the frequency of depressive symptoms, anxiety, and suicidal thoughts in students and non-students enrolled in the same study in France and during the same key periods of the COVID-19 epidemic. Using a repeated cross-sectional design, we collected data from a sample of 3783 participants in the CONFINS study during three recruitment waves between March 2020 and January 2021. Multivariate logistic regression models, adjusted for potential confounding factors, showed that students were more likely to have high scores of depressive symptoms and anxiety more frequently than non-students. These differences were particularly strong during the first (depressive symptoms: adjusted odds ratio aOR 1.59, 95% CI 1.22–2.08; anxiety: aOR 1.63, 95% CI 1.22–2.18) and second lockdowns (depressive symptoms: aOR 1.80, 95% CI 1.04–3.12; anxiety: aOR 2.25, 95% CI 1.24–4.10). These findings suggest that the restrictive measures—lockdown and curfew—have an alarmingly stronger negative impact on students than on non-students and underline the frailty of students’ mental health and the need to pay greater attention to this population in this epidemic-related context.

## Introduction

Even before the COVID-19 pandemic, it was known that college students have a high prevalence of mental health problems^1,2^. In France, as in most high-income countries, suicide is the second-leading cause of death among individuals aged 15 to 25 years, and a national student cohort has reported high rates of depression, anxiety, and suicidal thoughts^3^.

In the first stages of the epidemic, France was one of the most affected countries in the world in number of cases and deaths^4,5^, leading to an early lockdown in March 2020, followed by another lockdown period in October 2020. The negative impact of the COVID-19 epidemic and lockdown on mental health has been reported in some population-based studies^6–8^, some including students^9,10^. A recent meta-analysis including 27 studies constituting 90,879 college students in 15 countries reported 39.4% having anxiety and 31.2% having depression during the COVID-19 epidemic^11,12^.

To our knowledge, most of these studies were conducted in the initial months of the pandemic and, to date, none of them has presented some prevalence of mental health problems among students during the second lockdown in France. It is, however, uncertain whether students are indeed at higher risk of mental health disturbances than non-students or if they are differentially impacted by lockdown periods over time. Our objective was to compare the frequency of depressive symptoms, anxiety, and suicidal thoughts in students and non-students enrolled in the same study, in key periods of the COVID-19 epidemic from March 2020 to January 2021 in France.

## Methods

### Design, study population, and data source

Our study sample comprised participants in the ongoing web-based CONFINS cohort (www.confins.org), a prospective population-based study launched in March 2020 to address the psychological impact of COVID-19. Participants were recruited via advertisement in traditional and social media. To be eligible, subjects had to be aged 18 or older (without any upper age bar) and to have been confined in France. All participants provided an on-line informed consent. Using a repeated cross-sectional design, data collection occurred during three recruitment periods corresponding to key periods of the COVID-19 epidemic in France: (1) period 1: the first national lockdown (17th March–11th May, 2020); (2) period 2: no lockdown restrictions (12th May–27th October, 2020); and (3) period 3: the second national lockdown and curfew (28th October, 2020–25th January, 2021).

### Measures

*Depressive symptoms:* participants completed the French version of the 9-item Patient Health Questionnaire (PHQ-9), which is a reliable, valid measure of depression severity^13,14^. We used a validated cut-off of 10 to define the presence of depressive symptoms^15^.

*Anxiety symptoms:* participants completed the French version of the Generalized Anxiety Disorder-7 scale (GAD-7), a validated brief measure for assessing generalized anxiety symptoms^16,17^. We used a validated cut-off score of 10 to define the presence of anxiety symptoms^16^.

*Suicidal thoughts:* the questionnaire included a single-item question about suicidal thoughts during the last 7 days, as follows: “During the past 7 days, how often have you thought of attempting suicide (had suicidal ideation)?” Participants selected one of three possible responses: (1) no suicidal thoughts, (2) occasional suicidal thoughts, and (3) frequent suicidal thoughts. Occasional or frequent suicidal thoughts were considered together in this analysis.

*Covariates:* the following self-reported covariates were considered in the analyses: age, gender (male, female, other), marital status (single, married, or in a couple), education level (university studies or not), and psychiatric disease history (yes/no).

### Statistical analyses

We first described the overall study sample and grouped participants according to their student status and inclusion period. The continuous variable “age” is expressed as the mean ± standard error. Categorical variables are described as the count and proportion (%). The Kruskal–Wallis test was used to compare distributions of age in the groups (students or not). Proportions were compared using the chi-square test. To compare the frequency of mental health outcomes between students and non-students in each period, we used logistic regression models. The results are expressed as adjusted odds ratios (aORs) with 95% confidence intervals (CIs). Model convergences were checked. The assumption of linearity of the logit was tested for the continuous variable “age” in each model. The fully adjusted analyses took into account all selected covariates. In each model, to account for missing information on covariates, namely psychiatric disease history, we used the multiple imputation-by-chained equation method. Briefly, we performed 10 imputations and averaged the variable estimates to produce a mean estimate. Finally, we verified that the relative efficiency of the imputation for each variable was greater than 95%.

All analyses were performed using SAS version 9.4. Two-sided P values < 0.05 were considered statistically significant.

### Ethics approval

The study follows the principles of the Declaration of Helsinki, and the collection, storage, and analysis of the data comply with the General Data Protection Regulation (EU GDPR). The study was approved by the French Committee for the Protection of Individuals (Comité de Protection des Personnes—CPP IDF X, nr. 46-2020) and by the National Commission on Informatics and Liberty (Commission Nationale de l'Informatique et des Libertes) CNIL, nr. MLD/MFI/AR205600).

### Consent to participate

Students were informed of the nature and purpose of the study and provided on-line consent.

## Results

Among the 3783 participants included, 66.6% (n = 2518; 59.1% students) were recruited during period 1 (the first lockdown), 21.4% (n = 811; 63.6% students) during period 2 (no lockdown), and 12.0% (n = 454; 73.4% students) during period 3 (the second lockdown and curfew). Table 1 shows the characteristics of the study sample overall and according to the student status and the inclusion period. The mean age of the entire sample was 29.2 years (SD ± 11.7): 38.4 years (SD ± 13.9) for the non-students and 23.4 years (SD ± 3.9) for the students. About three-quarters were female (n = 2971; 78.6%) and about one participant in five reported psychiatric disease history.

**Table 1:** Characteristics of the study sample overall and according to the student status and the inclusion period, CONFINS study (2020–2021).

	All sample (n = 3783)			First lockdown (n = 2518; 66.6%)			No-lockdown period (n = 811; 21.4%)			Second lockdown (n = 454; 12.0%)		
	Total	Non-students (n = 1470; 38.9%)	Students (n = 2313; 61.1%)	Total	Non-students (n = 1031; 41.0%)	Students (n = 1487; 59.1%)	Total	Non-students (n = 295; 36.4%)	Students (n = 516; 63.6%)	Total	Non-students (n = 144; 26.6%)	Students (n = 310; 73.4%)
**Age** mean (SD)	29.2 (11.7)	**38.4 (13.9)**	**23.4 (3.9)**	30.1 (12.6)	**40.0 (14.2)**	**23.3 (3.9)**	27.8 (9.6)	**34.6 (12.1)**	**23.9 (3.8)**	26.9 (9.8)	**35.2 (12.9)**	**23.1 (3.9)**
**Gender**												
Men	20.8 (785)	**23.4 (344)**	**19.1 (441)**	21.7 (547)	**24.0 (247)**	**20.2 (300)**	19.1 (155)	20.7 (61)	18.2 (94)	18.3 (83)	25.0 (36)	15.2 (47)
Women	78.6 (2971)	**76.1 (1119)**	**80.1 (1853)**	77.6 (1954)	**75.6 (779)**	**79.0 (1175)**	80.5 (653)	79.0 (233)	81.4 (420)	80.2 (364)	74.3 (107)	82.9 (257)
Other	0.7 (27)	**0.5 (7)**	**0.9 (20)**	0.7 (17)	**0.5 (5)**	**0.8 (12)**	0.4 (3)	0.3 (1)	0.4 (2)	1.6 (7)	0.7 (1)	1.9 (6)
**Marital status**												
Married or in a couple	56.2 (2125)	**69.2 (1017)**	**47.9 (1108)**	56.5 (1422)	**69.3 (714)**	**47.6 (708)**	57.3 (465)	**68.8 (203)**	**50.8 (262)**	52.4 (238)	**30.6 (44)**	**55.5 (172)**
Single	43.8 (1658)	**30.8 (453)**	**52.1 (1205)**	43.5 (1096)	**30.8 (317)**	**52.4 (779)**	42.7 (346)	**31.2 (92)**	**49.2 (254)**	47.6 (216)	**69.4 (100)**	**44.5 (138)**
**University studies**												
	92.5 (3498)	**80.6 (1185)**	**100.0 (2313)**	90.7 (2283)	**77.2 (796)**	**100.0 (1487)**	94.5 (766)	**84.8 (250)**	**100.0 (516)**	98.9 (449)	**96.5 (139)**	**100.0 (310)**
**Concerns about financial situation**												
Important to very important	19.6 (725)	**16.7 (242)**	**21.4 (483)**	20.3 (502)	**17.5 (178)**	**22.3 (324)**	16.9 (134)	**14.9 (43)**	**18.1 (91)**	19.9 (89)	14.9 (21)	22.2 (68)
Few or no	43.8 (1658)	**83.3 (1204)**	**78.6 (1778)**	79.7 (1967)	**82.5 (838)**	**77.7 (1129)**	83.1 (657)	**85.1 (246)**	**81.9 (411)**	80.1 (358)	85.1 (120)	77.8 (238)
**Psychiatric disease history** (missing = 657)												
	21.0 (656)	**21.3 (247)**	**20.8 (409)**	23.1 (443)	23.7 (176)	22.7 (267)	18.4 (134)	20.1 (54)	17.4 (80)	20.4 (79)	**13.0 (17)**	**24.2 (62)**
**Depressive symptoms**												
	30.2 (1141)	**20.1 (295)**	**36.6 (846)**	29.1 (733)	**18.7 (193)**	**36.3 (540)**	25.8 (203)	21.4 (63)	27.1 (140)	45.2 (205)	**27.1 (39)**	**53.6 (166)**
**Anxiety**												
	23.4 (884)	**16.9 (249)**	**27.5 (635)**	22.1 (557)	**15.9 (164)**	**26.4 (393)**	20.0 (59)	21.1 (109)	20.1 (109)	35.0 (159)	**18.1 (26)**	**42.9 (133)**
**Suicidal thoughts** (missing = 740)												
	10.9 (331)	**7.9 (89)**	**12.7 (242)**	10.1 (195)	**7.3 (54)**	**11.9 (142)**	10.5 (76)	8.3 (22)	11.8 (54)	15.5 (60)	11.1 (14)	17.6 (46)

All data presented as %(*N*) unless otherwise noted (SD: Standard deviation).

Two-sided *P* values < 0.05 were considered statistically significant based on Kruskal–Wallis test for continuous variable and chi-square test for categorical variables comparing students and non-students (results in bold).

The majority of the non-students (n = 1185; 80.6%) declared a university education level. This group was more likely to be married or in a couple than were students (69.2% vs. 47.9%). Important concerns about financial situation were more frequent among students than among non-students (21.4% vs. 1.7%). The patterns of sociodemographic characteristics of each group were stable, whatever the inclusion period.

**Figure 1:**
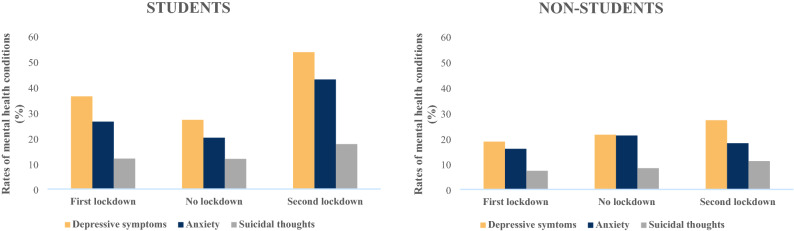
Rates of depressive and anxious symptoms as well as suicidal thoughts (%) among students and non-students according to key periods of the COVID-19 epidemic in France, CONFINS study (2020–2021).

In the overall sample, students had higher rates of mental health symptoms than non-students, including depressive symptoms (36.6% vs. 20.1%), anxiety (27.5% vs. 16.9%), and suicidal thoughts (12.7% vs. 7.9%). Moreover, we observed a dramatic differential pattern between students and non-students according to the period (Fig. 1). Specifically, among students, there was a higher frequency of depressive symptoms and anxiety during the first and second lockdowns compared with the no-lockdown period. Among non-students, the rates of mental health conditions were relatively stable over time. When comparing both groups, the frequency of depressive symptoms during the first lockdown was nearly twice as high in students as in non-students (36.3% vs. 18.7%; P < 0.0001). In period 2 with no lockdown restrictions, the difference was attenuated (27.1% in students vs. 21.4%; P = 0.0677) and non-significantly different. However, during the second lockdown, more than one-half of the students had depressive symptoms, compared with about one-quarter of the non-students (53.6% vs. 27.1%; P < 0.0001).

Multivariate analyses confirmed significant variations in the rates of mental health conditions over time in the student population, but not in the non-student population (Table 2). Adjusting for age, gender, psychiatric disease history, education level, and marital status, students had an approximately 60% increase in risk of depressive symptoms (aOR 1.59, 95% CI 1.22–2.08) and anxiety (aOR 1.63, 95% CI 1.22–2.18) compared with non-students during the first lockdown. During the second lockdown, the point estimates were even higher for both depressive symptoms (aOR 1.80, 95% CI 1.04–3.12) and anxiety (aOR 2.25, 95% CI 1.24–4.10). However, in the no-lockdown period, there was no difference between the two groups. There was no statistically significant difference between groups regarding suicidal thoughts at any period.

**Table 2:** Association between college student status and depressive symptoms (n = 3783), anxiety (n = 3783), suicidal thoughts (n = 3043) according to key periods of the COVID-19 epidemic in France, CONFINS study (2020–2021).

	First lockdown		No-lockdown period		Second lockdown	
	OR	95% CI	OR	95% CI	OR	95% CI
**Model 1**						
Depressive symptoms	1.44	[1.10–1.89]	1.24	[0.81–1.89]	2.13	[1.24–3.66]
Anxiety	1.52	[1.14–2.05]	0.82	[0.52–1.29]	2.65	[1.46–4.82]
Suicidal thoughts	1.59	[0.98–2.56]	1.35	[0.72–2.55]	1.24	[0.56–2.79]
**Model 2**						
Depressive symptoms	1.59	[1.22–2.08]	1.17	[0.76–1.80]	1.80	[1.04–3.12]
Anxiety	1.63	[1.22–2.18]	0.94	[0.59–1.48]	2.25	[1.24–4.10]
Suicidal thoughts	1.21	[0.91–1.60]	0.82	[0.42–1.61]	0.99	[0.53–1.85]

Model 1 adjusted for age and gender; Model 2 adjusted for age, gender, psychiatric disease history, education level, marital status and after multiple imputation on psychiatric disease history.

*OR* odds ratio, *CI* confidence interval.

## Discussion

In this study of 3783 participants, we found that students were more likely than non-students to have high scores for depressive symptoms and anxiety during the COVID-19 pandemic. This pattern was particularly strong during lockdown periods, suggesting that students are a more fragile group under these conditions. While the period with no lockdown restrictions was accompanied by a clear improvement in mental health outcomes in students, the second lockdown seemed to have an even stronger effect on these mental outcomes in students.

In the early stages of the epidemic, previous studies among students reported similar high prevalences of depressive and anxiety symptoms in other countries^18–20^ and in France^12,21,22^. Our findings could be explained by the lack of support, social isolation^23,24^, and well-known vulnerability of students to mental health problems^1^ that were likely exacerbated by the COVID-19 pandemic and lockdown restrictions. Since the beginning of the epidemic, students have had to modify their living and working habits and adapt to remote pedagogical practices. Most universities have been closed, and the loss of jobs or concerns about health may have contributed to the stress and declining mental health of students^25^. Moreover, the duration of the epidemic and the restrictions may also have led to a feeling of weariness and aggravated initial situations of vulnerability linked to the students’ financial situations or social isolation.

This study has some important strengths, including the large sample of participants which allowed a comparison between students and non-students in different key periods of the COVID-19 pandemic. Students and non-students were recruited using the same strategy and differed mainly in mean age and marital status. Depressive and anxiety symptoms were measured using validated scales that have well-known appropriate psychometric properties. Despite these strengths, some limitations should be considered. First, our study design was cross-sectional. Therefore, these findings do not reflect the evolution of mental conditions in our population but the differences between samples of individuals recruited at different times during the epidemic. Second, the participants were volunteers, which may have introduced a self-selection bias. However, as mentioned above, recruitment strategies were maintained during the entire duration of the study and were based on the same messages for both students and non-students. Selection bias, if any, should have affected both groups similarly during the various recruitment phases. Third, because of the potential self-selection and the particularities of our sample, it is not possible to generalize our conclusions to all students and non-students in France or elsewhere. Nevertheless, we believe that the differences we observed between students and non-students may be similar in other settings. Fourth, the self-reported questionnaires could lead to information and recall bias, particularly if participants under-reported their frequency of mental health conditions due to concerns about social desirability. However, such under-reporting is likely to be reduced by the use of an online questionnaire and it is unlikely that it explains the large differences observed between students and non-students. Fifth, although we have included some major covariates in our multivariate models, unmeasured confounding factors could remain and partly explain the association between student status and mental health conditions.

In conclusion, our findings provide new insights into the mental health of university students during the COVID-19 epidemic. These results suggesting that the restrictive measures of lockdown and curfew have an alarmingly stronger negative impact on students than on non-students, underline the frailty of students’ mental health, and show that greater attention should be given to this population. Future studies should implement appropriate interventions to reduce this impact and promote the mental health of students, for example by improving posttraumatic growth or stress management. Given that the COVID-19 epidemic and some restrictions are set to continue for some time and that long-term consequences to mental health are likely to occur, it is crucial that universities and public health systems consider long-term strategies in terms of screening and help to mitigate mental health disorders in students during the pandemic.

## Data Availability

The datasets used and/or analyzed during the current study are available from the corresponding author on reasonable request.
